# Acceptance and Compassion-Based Therapy Targeting Shame in Body Dysmorphic Disorder: A Multiple Baseline Study

**DOI:** 10.1177/01454455221129989

**Published:** 2022-11-13

**Authors:** Johanna Linde, Jason B. Luoma, Christian Rück, Jonas Ramnerö, Tobias Lundgren

**Affiliations:** 1 Centre for Psychiatry Research, Department of Clinical Neuroscience, Karolinska Institutet, Stockholm, Sweden; 2 Portland Psychotherapy Clinic, Research, & Training Center, OR, USA

**Keywords:** acceptance and commitment therapy, body dysmorphic disorder, compassion, shame, treatment

## Abstract

Shame is considered central in body dysmorphic disorder (BDD) and empirical accounts highlight the link between shame and BDD symptoms as well as common negative psychosocial effects of the disorder, yet there is a lack of interventions addressing shame in this context. In the past decade, Acceptance and commitment therapy (ACT) and interventions that foster self-compassion have shown promise for reducing the negative effects of shame in a range of clinical problems. The aim of the present study was to develop and evaluate an acceptance and compassion-based treatment specifically targeting shame in BDD. Using a randomized nonconcurrent multiple baseline design, the 12-session intervention, ACT with Compassion (ACTwC), was examined in a psychiatric outpatient sample of five adults diagnosed with BDD. The daily ratings showed marked reductions in BDD-behaviors and self-criticism at posttreatment for four of five participants, while three participants demonstrated decreases in body shame compared to baseline. Improvements were maintained at 6-months follow-up. The intervention also led to reliable long-term improvements in general shame, overall BDD-symptoms, depressive symptoms, and quality of life for four of five participants. All treatment responders showed significant gains in psychological flexibility and self-compassion. Participants reported high credibility and satisfaction with the treatment. These preliminary results suggest that ACTwC may be a promising approach to treating shame in BDD, worthy of further investigation.

## Introduction

Body dysmorphic disorder (BDD), characterized by a preoccupation with a perceived flaw in appearance (American Psychiatric Association, 2013), is a debilitating psychiatric disorder, associated with decreased quality of life (Phillips, 2000) and impairment in interpersonal and social functioning in nearly all patients (Phillips & Diaz, 1997; Phillips, Didie, Menard, Pagano, et al., 2006). Comorbid major depressive disorder occurs at some point in 55% to 83% of individuals with BDD (Phillips, Didie, & Menard, 2006; Phillips, Menard, et al., 2005) and about one quarter have made a suicide attempt (Phillips, 2007; Phillips, Coles, et al., 2005). Current state-of-the-art treatment for BDD, Cognitive Behavioral Therapy (CBT), is recognized as effective. However, a substantial proportion of individuals with BDD (46%–60%) treated with CBT are non-responders (for review see Harrison et al., 2016). If left untreated the disorder has a chronic course (Phillips et al., 2013). Thus, there is an urgent need for further research on effective interventions for BDD.

One way to improve treatment outcomes for BDD may be to target shame, an emotion considered central in this disorder (Weingarden & Renshaw, 2014). A growing body of research suggests that shame considerably contributes to the symptoms and adverse outcomes of BDD (Weingarden et al., 2016, 2017), yet little is known about effective interventions for reducing the negative effects of shame in this context. The present study provides a first step in filling this gap, by investigating the effects of a psychological intervention especially developed to target shame in BDD.

Shame refers to the affective experience of perceiving the self as intrinsically flawed and socially undesirable (Lewis, 1971). It involves self-criticism, including global negative self-evaluations for perceived flaws or shortcomings, and action urges to “disappear” or hide (Tangney & Dearing, 2002). In conceptualizations of BDD, the role of shame in the development and maintenance of BDD has been emphasized. Veale and Gilbert (2014) have proposed that shame drives problematic behaviors common in BDD, such as checking and camouflaging appearances, comparing self with others and social avoidance.

Accumulating research points to considerable negative effects of shame in BDD. Shame contributes significantly to increased overall BDD-symptoms and commonly co-occurring negative psychosocial consequences, such as limited functioning, hopelessness, depression, and suicidal thoughts (Weingarden et al., 2016, 2017). Considering the high rates of adverse psychosocial outcomes among people with BDD, these findings suggest that shame may indeed be an important target in BDD treatment.

Even though research shows a link between shame and severe adverse outcomes in BDD, there are yet no evaluations of interventions specifically targeting shame in this disorder. One reason for this is perhaps that CBT, the most established psychological treatment method for BDD, is focused on treating maladaptive cognitions and diagnosis-specific symptoms, rather than shame. However, when treating people who are highly shame-prone and self-critical, theorists suggests that shame needs to be directly targeted in treatment (Tangney & Dearing, 2002).

In the past decade, evidence has emerged supporting the use of interventions developed in more recent “third-wave therapies” building on the CBT tradition, for conditions where shame is a prominent feature. Treatment approaches such as Acceptance and commitment therapy (ACT) and Compassion focused therapy (CFT), have shown potential to reduce shame and associated problems in a range of clinical conditions; for example, binge eating disorder (Pinto-Gouveia et al., 2019), eating disorders (Goss & Allan, 2014), alcohol misuse (Luoma et al., 2012) as well as mood and personality disorders (Cuppage et al., 2018; Gilbert & Procter, 2006).

ACT is proposed to be an effective approach for reducing negative effects of shame due to the treatment’s potential to decrease experiential avoidance and increase psychological flexibility, including weakening the influence of self-critical thoughts (Luoma & Platt, 2015). Experiential avoidance refers to the tendency to avoid difficult private experiences (e.g., shame), even when it leads to both short- and long-term problems. Empirically, experiential avoidance has been associated with higher levels of shame (Luoma et al., 2006; Mitmansgruber et al., 2009) and shown to mediate the impact of shame experiences on depression symptoms (Carvalho et al., 2013). Compared to healthy controls, individuals with BDD present with significantly greater experiential avoidance (Wilson et al., 2014), suggesting an ACT approach could be useful for this group of patients. In order to decrease experiential avoidance, ACT promotes psychological flexibility, which is defined as being able to nonjudgmentally and willingly notice private experiences, direct attention to the present moment while engaging in actions that are personally meaningful (Hayes et al., 1999).

Another promising approach for alleviating detrimental effects of shame and self-criticism is to build self-compassion (Leaviss & Uttley, 2015). Self-compassion is about relating to oneself in a specific way that involves being mindfully aware of one’s own suffering and responding to oneself with a caregiving repertoire of kindness, warmth, and goodwill because of the suffering (Neff, 2003). Compassion-focused therapy (CFT) grew out of the clinical observation that individuals high in shame and self-criticism find it difficult to generate affiliative, warm feelings toward the self (Gilbert, 2005). A central task in CFT is to foster a person’s felt sense of self-kindness and cultivate these caregiving repertoires toward the self. Empirically, self-compassion has been recognized as a protective factor that may buffer the negative effects of shame related to the body. There are consistent empirical demonstrations of a negative association between self-compassion and body shame (see Braun et al., 2016 for review). Further, self-compassion has been found to moderate the relationship between body-related shame and depression among women (Sick et al., 2020) and is implicated as a protective factor against poor body image (Braun et al., 2016).

Based on advances in the research field on shame and previous research on BDD, we have developed a psychological treatment intervention targeting shame in BDD. The manualized treatment, ACT with Compassion (ACTwC) for BDD, integrates ACT with elements from CFT and knowledge on BDD. ACTwC for BDD, is designed to target shame and self-criticism and increase psychological flexibility and self-compassion with the overarching goal of increasing quality of life in patients suffering from BDD. An earlier version of this treatment that did not include elements focused on compassion and shame (ACT for BDD) was tested in a group therapy format with 21 patients with BDD (Linde et al., 2015). Initial results of this open trial were promising, with large reductions in BDD-symptoms (*d* = 1.93, *p* .001), reliable increases in psychological flexibility and quality of life, and a 79% treatment response rate.

The purpose of the present study was to evaluate the effect and feasibility of a psychological treatment intervention directly targeting shame in BDD. In this early stage of evaluating a new treatment approach to BDD, our objectives were to investigate; (a) preliminary effectiveness and (b) patient’s acceptance of and satisfaction with the treatment. Using a multiple baseline design, the treatment’s effect on the primary outcomes of body shame, self-criticism, and BDD behaviors, was evaluated. Secondary outcomes were overall symptoms of BDD, depression, quality of life, self-compassion, and psychological flexibility. Treatment feasibility was evaluated by investigating patient’s perceived credibility and expectancy as well as satisfaction with the intervention. We aimed to evaluate the feasibility of the treatment in a real-world context and therefore chose to conduct the study in a psychiatric outpatient clinic.

## Method

### Participants and Setting

Participants were recruited and treated at a psychiatric outpatient clinic in Stockholm, Sweden, specialized in treatment for OCD-spectrum disorders. Five individuals with BDD as their primary diagnosis, assessed by a psychiatrist and/or a clinical psychologist at the clinic, participated in the study. Diagnoses were based on the obsessive–compulsive and related disorders module of the Structured Clinical Interview for DSM-5 (First et al., 2015), and the Mini-International Neuropsychiatric Interview 7 (MINI; Sheehan et al., 1998). The assessors confirmed eligibility and obtained consent for participation.

Inclusion criteria were: (a) 18 years or older; (b) diagnosis of BDD according to DSM-5 with BDD-symptoms of at least moderate severity (BDD-YBOCS score ≥23); and (c) elevated shame (ISS score >40). Exclusion criteria were: (a) severe suicidal ideation and intent (as defined by score >4 on item 9 of MADRS-S), (b) lifetime diagnoses of bipolar disorder, (c) psychosis, (d) autism spectrum disorder or (e) a severe personality disorder (such as borderline personality disorder with self-harm), (f) current substance dependence, (g) psychotropic medication changes within 12 weeks prior to treatment, (h) completed CBT for BDD within 12 months, or (i) concurrent psychotherapy. Participants who were on a stable dose of psychotropic drugs were asked to not make changes to their pharmacological treatment during the study period. Participant characteristics are presented in Table 1.

**Table 1.** Participant Characteristics.

	Age	Gender	Marital status	Education	Employment status	Main BDD concerns	Main BDD behaviors	Comorbidity	Years since debut of BDD	Medication
P1	23	F	IR	High school	PL (FT)	Proportions of features	Comparing, mirror gazing, measuring bodyparts	None	9	None
P2	27	F	IR	University	ST	Nose, eyes, shape of head, hair	Comparing, camouflaging, checking, social avoidance	MDD, agoraphobia, dermatillomania	12	SSRI
P4	25	F	S	University	PT	Facial features, hair, shoulders, arms	Camouflaging, styling, comparing, checking	MDD, SAD, GAD	10	SSRI
P5	23	M	IR	High school	ST; PT	Hair	Camouflaging, mirror gazing, styling	ADHD, ASPD, OCD	9	SSRI
P7	24	M	S	University	ST	Hair, skin	Camouflaging, styling, checking, social avoidance	MDD, SAD	7	SSRI

*Note.* P = participant; F = female; M = male; IR = in relationship; S = single; PL = parental leave; FT = employed full-time; ST = student; PT = employed part-time; MDD = major depressive disorder; SAD = social anxiety disorder; GAD = generalized anxiety disorder; ADHD = attention deficit hyperactivity disorder; ASPD = antisocial personality disorder; OCD = obsessive compulsive disorder.

Seven consecutive participants meeting inclusion and not exclusion criteria were invited to take part in the study. One participant was withdrawn from the study before initiating treatment due to increased suicidality requiring a higher level of care at an inpatient clinic. Another patient dropped out early during treatment when face-to-face sessions at the clinic changed to video sessions due to Covid-19. The two non-completers did not have sufficient data to be included in the final analyses, leaving a sample of five treatment completers. Non-completers did not differ distinctly from completers in terms of demographics, initial symptom severity, comorbidity, type of BDD concerns or BDD behaviors.

### Study Design

To investigate the effectiveness of the intervention for reducing body shame, self-criticism, and BDD-behaviors, a randomized, nonconcurrent, multiple baseline across participants design was used (Barlow et al., 2009; Kazdin, 2011). Consistent with single-case design principles, the primary outcomes, were assessed repeatedly over time with the aim of evaluating whether changes occurred after treatment was introduced.

Following enrollment, participants were randomized to baseline assessment phases of different lengths, between 2 and 6 weeks, using randomly drawn sealed envelopes. Participants completed daily self-report measures via a secure Internet-based platform throughout the baseline and subsequent 12-session treatment phase and at 6-months follow-ups. In addition to the daily measures, standardized outcome measures were used to evaluate the secondary outcomes at baseline, mid-treatment, posttreatment, and at 6-month follow-up.

A second aim was to investigate feasibility of this new treatment approach to BDD. We assessed credibility of treatment and patient’s expectancy for improvement (after the third session) and satisfaction with the treatment (after treatment completion).

Prior to data collection, the study was registered and approved by the regional ethics committee (Registration number 2018/2374-31).

### Measures

*Structured Clinical Interview for DSM 5—Research Version (SCID-5-RV), module G* (First et al., 2015). The SCID-5-RV is a semi-structured, clinician-administered interview designed to diagnose disorders according to the DSM-5. For the purposes of the present study, only module G (obsessive-compulsive and related disorders) was utilized.

*The MINI International Neuropsychiatric Interview 7.0* (Sheehan et al., 1998). The MINI is a relatively short, but psychometrically sound, structured interview used to assess 17 common Axis I disorders. It was used to assess comorbid conditions, and to identify conditions that merited exclusion from this study. The MINI was administered by psychiatrists with extensive training in administering this instrument.

#### Primary outcomes

The primary outcome measure was the daily self-monitoring of BDD-related behaviors and the level of body shame and self-criticism experienced. Before baseline, the concepts of BDD-behaviors, shame, and self-criticism were explained and participants were shown how to fill out the daily assessment.

##### Daily time spent on BDD-behaviors

The daily record sheet asked participants to record the time spent on BDD-related behaviors, categorized in checking, comparing, camouflaging, and avoidance behaviors (e.g., “Number of minutes spent on camouflaging”). The sum of each participants’ daily BDD-behaviors is presented in the results section.

##### Daily ratings of body shame and self-criticism

Along with the BDD-behaviors, participants were asked to rate their degree of (1) body shame (“On a 0–10 scale, where 0 = no shame at all, and 10 = the most shame ever, to what extent have you felt ashamed because of your appearance today?”) and (2) self-criticism (“On a 0–10 scale, where 0 = not self-critical at all, and 10 = the most self-critical ever, how self-critical have you been today?”).

#### Secondary outcomes

##### BDD symptoms

The Yale-Brown Obsessive Compulsive Scale Modified for BDD (BDD-YBOCS; Phillips et al., 1997) was used to rate the overall severity of BDD during the previous week. It consists of 12 items and is the most widely used measure for assessing BDD symptoms. The range is 0 to 48 where a higher score indicates greater severity. BDD-YBOCS has high internal reliability (α = .92; Phillips et al., 2014).

##### General shame

The Internalized Shame Scale (ISS; Cook, 1988), consists of 24 items measuring participants’ level of internalized shame. Respondents rate frequency of various shame experiences (e.g., “I feel intensely inadequate and full of self doubt”). Total scores range from 0 to 96. It has demonstrated good psychometric properties (Cook, 1988).

##### Self-compassion and self-criticism

Self-compassion was measured with the 12-item Self-Compassion Scale-Short Form (SCS-SF; Raes et al., 2011), which has been found to show near-perfect correlation (*r* ≥ .97) with the full 26-item Self-Compassion Scale (SCS; Neff, 2003). The scale was developed to assess the extent to which individuals relate to themselves compassionately at times of distress and disappointment. Total SCS-SF score is the mean of six subscales and ranges from 1 to 5, with higher scores indicating greater self-compassion. The SCS-SF contains a self-judgment subscale; here considered to be equivalent to self-criticism. Neff (2003) conceptualizes self-judgment as being at the opposite end of a continuum from self-kindness and this subscale is therefore reverse scored when used to contribute to an overall measure of self-compassion. The items of the self-judgment subscale focus on being disapproving and intolerant about one’s flaws and being tough on oneself at times of suffering.

##### Depression

The Montgomery-Åsberg Depression Rating Scale (MADRS-S; Svanborg & Åsberg, 1994) is a widely used self-report depression scale. This scale consists of nine items, each measuring a different symptom (mood, feelings of unease, sleep, appetite, ability to concentrate, initiative, emotional involvement, pessimism, and suicidal ideation) on a 7-point scale with a total score ranging from 0 to 54. It was used to assess depressive symptomatology experienced over the previous 3 days. The MADRS-S has good to excellent test-retest reliability (*r* = .80–.94) and correlates (*r* = .87) with the Beck Depression Inventory, indicating acceptable convergent validity (Svanborg & Åsberg, 2001).

##### Quality of life

The Quality of Life Inventory (QOLI; Frisch et al., 1992) is a self-report questionnaire that assesses quality of life based on the degree of satisfaction in 16 different life domains (e.g., work and social life). For each domain, the respondent is asked to rate the level of importance on a 3-point scale (0–2) and the degree of satisfaction on a 6-point scale (−3 to +3). By multiplying importance by satisfaction, a value from −6 to +6 for each domain is obtained. The total score is the average score of domains rated as important or very important. The QOLI has shown good test-retest reliability and high internal consistency (Frisch et al., 1992). QOLI was administered at pre, post, and follow up.

##### Psychological flexibility

The Body Dysmorphic Disorder Acceptance and Action Questionnaire (BDD-AAQ) is an investigator created 12-item measure of willingness to accept undesirable thoughts and feelings associated with one’s appearance, while acting in a way that is congruent with values and goals. All items are reverse scored and are summed to produce a total score. Higher scores indicate greater psychological flexibility. The scale (included in Supplemental Material) was adapted for a BDD population from the Body Image-Acceptance and Action Questionnaire (BI-AAQ; Sandoz et al., 2013).

##### Cognitive fusion

The Cognitive Fusion Questionnaire–7 (CFQ; Gillanders et al., 2014) The CFQ is a 7-item measure of the process cognitive fusion: the degree to which people believe in the content of their thinking versus having some degree of distance or objectivity from thoughts. Items are rated on a Likert scale of 1 (“never true”) to 7 (“always true”) and are summed. Lower scores reflect greater defusion. Gillanders et al. (2014) found it has acceptable internal consistency (.88) and test-retest reliability (.80).

##### Treatment credibility

Credibility/Expectancy Questionnaire (CEQ; Borkovec & Nau, 1972) measures the credibility of treatment and expectancy for improvement on a 10-point Likert-type scale. Participants perceived credibility and expectancy of ACTwC was assessed through items 1 (“How logical does the treatment offered to you seem?”) and 2 (“How confident are you that this treatment will be successful in treating your BDD?”). Higher scores indicate greater treatment credibility and expectancy for improvement.

##### Treatment satisfaction

Treatment satisfaction was assessed with Client Satisfaction Questionnaire (CSQ-8; Nguyen et al., 1983) which comprises eight items, assessing satisfaction with a specific healthcare or counseling service.

### Intervention

The treatment, ACTwC, was designed to target shame and self-criticism and increase self-compassion, psychological flexibility, and quality of life in patients suffering from BDD. A manual to guide each treatment session was developed based on integrating research on BDD with theory and techniques from ACT and compassion-focused therapies (see Supplemental Material for a session-by-session overview of the protocol).

The intervention consisted of 12 individual therapy sessions, around 60 minutes each; including psychoeducation on the treatment concepts (e.g., conceptualization of BDD from an ACT and compassion perspective); building skills in present-centered, nonjudgmental awareness, especially of one’s own suffering (e.g., through basic mindfulness training); experiential exercises (aiming at fostering self-compassionate self-talk, defusion, acceptance, and perspective taking abilities); training in acknowledging, eliciting, and regulating previously avoided emotions of shame (e.g., exposure to internalized shame through chair-work-techniques); practicing self-compassion in response to shame and self-criticism and promotion of value-guided actions in daily life.

Treatment was delivered by three clinical psychologists with previous experience in treating BDD patients. The first author, with expertise in BDD treatment, developed the treatment manual in collaboration with experts in ACT, treated two of the patients and also supervised the other two therapists weekly. Before delivering treatment, the therapists received approximately 3 hours of training in the manual. All treatment sessions were audio-recorded for weekly supervision and therapist adherence to the treatment manual. Sessions were divided into 10 minute modules that were chosen randomly for adherence checks. Adherence to the session content and structure as well as therapist adherence to ACT and compassion principles were evaluated by the treatment developer using a predefined adherence manual (available on request). Adherence level was rated as either sufficient, or not sufficient. The majority of the modules (90%) was judged as having been conducted in a sufficient manner.

### Data Analysis

Data analyses were conducted according to established guidelines for single-case experimental designs, with visual inspection of plotted data as the primary method (Barlow et al., 2009; Kazdin, 2011). The weekly average of each participant’s scores of BDD behaviors, body shame, and self-criticism were plotted in individual graphs. For missing data, the mean of the scores from the previous and following weeks were calculated. The graphed ratings were visually examined to evaluate the magnitude, rate of change, and data overlap across phases. The level and slope of the outcome variables during the treatment phase were compared against the baseline phase data, both within- and between-subjects. For statistical analysis we used Tau-U (Parker et al., 2011) to evaluate data nonoverlap between phases, controlling for baseline trend. The statistic reflects client improvement across the phases controlling for preexisting (baseline) improvement trend.

For the standardized outcome measures, 95% confidence intervals (CIs) were calculated for each participant’s change scores to evaluate the reliability of the change from pre- to posttreatment and change from pretreatment to 6-month follow-up. For each outcome measure, a standard error of the difference (Sdiff) was calculated, following the method developed by Jacobson and Truax (1991) for calculating reliable change. Sdiff represents the average change in score that would be expected on that measure by chance variation alone, between two measurement occasions. The Sdiff for each measure was then multiplied by 1.96 to create a 95% CI around each participant’s change score. When the CI does not include zero, the observed change can be considered a reliable change. CIs were not calculated for scores on the BDD-AAQ or the self-judgment subscale of the SCS due to lack of previous psychometric studies for these instruments.

The BDD-YBOCS scale was used to identify the number of participants who achieved *treatment response* and *symptom remission* from baseline to posttreatment and 6-months follow-up. We used the established criteria of a BDD-YBOCS reduction ≥30% to define treatment response and a score of ≤16 as cut-off for symptom remission (Fernández de la Cruz et al., 2021).

Feasibility data from the standardized measures of credibility, expectancy, and satisfaction with treatment is presented in descriptive form.

## Results

### BDD Symptoms

At the initial assessment, all five participants met the criteria for BDD according to DSM 5, with scores on the BDD-YBOCS indicating moderate to severe BDD symptom severity (*M* = 29.6, *SD* = 4.1, range 26–34).

**Figure 1.**
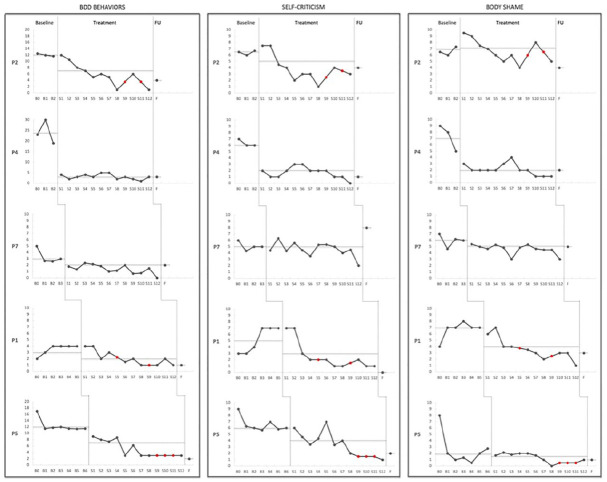
Individual ratings of BDD behaviors, self-criticism, and body shame throughout baseline, treatment, and during follow-up. *Note*. BDD behaviors reflect total amount of hours spent daily on BDD behaviors. Degree of self-criticism and body shame were rated on a 0 to 10 Likert scale. Means are represented by dashed lines for each phase. Red dots indicate imputed scores for missing data. Participants are presented in order of increasing baseline length. P = participant; B = baseline; S = session; F = 6 month follow-up.

Visual inspection of baseline data (Figure 1) indicates that BDD behavior scores were stable during baseline for all participants except for P4. The changes in level and trend after treatment was introduced indicate a consistent pattern of improvement across participants and by posttreatment, all had scores that did not overlap with their baseline scores. Changes in level were maintained 6 months posttreatment. As displayed in Table 2, Tau-U analysis showed significant reductions in BDD behaviors (*p* < .01) for all five participants between baseline and the intervention phase.

**Table 2.** Summary of Tau-U Analysis Comparing Baseline Phase With Treatment Phase Across Daily Measures.

BDD behaviors					Self-criticism				Body shame			
	Tau	*SD* Tau	*p* Value	90% CI	Tau	*SD* Tau	*p* Value	90% CI	Tau	*SD* Tau	*p* Value	90% CI
P1	−0.631	0.230	.006**	[−1, −0.252]	−0.648	0.230	.005**	[−1, −0.269]	−0.790	0.230	.001**	[−1, −0.411]
P2	−0.744	0.285	.009**	[−1, −0.275]	−0.578	0.285	.043*	[−1, −0.108]	−0.022	0.285	.938	[−0.492, 0.447]
P4	−0.994	0.345	.004**	[−1, −0.426]	−0.958	0.345	.006**	[−1, −0.390]	−0.897	0.345	.009**	[−1, −0.329]
P5	−0.816	0.161	.000**	[−1, −0.552]	−0.671	0.161	.000**	[−0.936, −0.406]	−0.072	0.161	.655	[−0.336, 0.193]
P7	−0.686	0.188	.000**	[−0.995, −0.377]	0.009	0.188	.963	[−0.300, 0.318]	−0.458	0.188	.015*	[−0.767, −0.149]

*Note. SD* = standard deviation; CI = confidence interval.

* *p* < .05. **p < .01.

Overall BDD symptoms reliably decreased for four of five participants (Table 3) with at least a 30% reduction in scores on the BDD-YBOCS indicating responder status. The four responders fell in the range of subclinical severity following treatment. P7 showed slight but nonsignificant improvement during treatment phase. At follow-up, all participants except for P7 maintained or extended their improvements and had achieved symptom remission (score of ≤16 on the BDD-YBOCS).

**Table 3.** Assessment and Change Scores With 95% CIs for BDD Severity, Shame, Depression, and Quality of Life.

BDD-YBOCS									ISS								
	Pre	Mid	Post	FU	Pre-post Δ (%)	Pre-FU Δ (%)	95% CI = CS ± 5.17^a^			Pre	Mid	Post	FU	Pre-post Δ (%)	Pre-FU Δ (%)	95% CI = CS ± 11.00^a^	
							Pre-post	Pre-FU								Pre-post	Pre-FU
P1	26	17	15	15	−42	−42	[−16.17, −5.83]*	[−16.17, −5.83]*	P1	68	60	47	51	−31	−25	[−32, −10]*	[−28, −6]*
P2	34	27	18	16	−47	−53	[−21.17, −10.83]*	[−23.17, −12.83]*	P2	78	86	66	54	−15	−31	[−23, −1]*	[−35, −13]*
P4	28	19	14	14	−50	−50	[−19.17, −8.83]*	[−19.17, −8.83]*	P4	75	80	77	42	3	−44	[−9, 13]	[−44, −22]*
P5	34		15	14	−56	−59	[−24.17, −13.83]*	[−25,17, −14.83]*	P5	58		16	20	−72	−66	[−53, −31]*	[−49, −27]*
P7	26	26	24	29	−8	12	[−7.17, 3.17]	[−2.17, 8.17]	P7	66	72	59	71	−11	8	[−18, 4]	[−6, 16]
MADRS-S									QOLI								
	Pre	Mid	Post	FU	Pre-post Δ (%)	Pre-FU Δ (%)	95% CI = CS ± 7.60^a^			Pre	Post	FU	Pre-post Δ (%)	Pre-FU Δ (%)	95% CI = CS ± 2.12^a^		
							Pre-post	Pre-FU							Pre-post	Pre-FU	
P1	33	8	7	5	−79	−85	[−33.6, −18.4]*	[−35.6, −20.4]*	P1	−2.88	1.94	0.07	167	102	[2.7, 6.94]*	[0.83, 5.07]*	
P2	35	15	7	14	−80	−60	[−35.6, −20.4]*	[−28.6, −13.4]*	P2	−2.6	3.13	2	220	177	[3.61, 7.85]*	[2.48, 6.72]*	
P4	27	22	19	3	−30	−89	[−15.6], −0.4]*	[−31.6, −16.4]*	P4	1.21	1.73	3.5	43	189	[−1.6, 2.64]	[0.17, 4.41]*	
P5	23		9	8	−61	−65	[−21.6, −6.4]*	[−22.6, −7.4]*	P5	0.73	2.93	3.73	301	411	[0.08, 4.32]*	[0.88, 5.12]*	
P7	27	25	23	30	−15	11	[−11.6, 3.6]	[−4.6, 10.6]	P7	−2.64	−3.23	−3.69	−22	−40	[−2.71, 1.53]	[−3.17, 1.07]	

*Note*. Negative change scores indicate decreases on a given measure, positive change scores indicate increases. CI = confidence interval; CS = change score; pre-post = change from pretreatment score to post-test (after Session 12); Pre-*FU* = change from pretreatment to 6 month follow-up; P = participant; BDD-YBOCS = The Yale-Brown Obsessive compulsive Scale Modified for BDD; ISS = Internalized Shame Scale; MADRS-S = The Montgomery-Åsberg Depression Rating Scale; QOLI = Quality of Life Inventory.

a The 1.96 × Sdiff value used for each measure to calculate the 95% CIs around each change score.

* Indicates improvement *p* < .05.

### Self-criticism

Visual inspection indicates that self-criticism was stable or worsened during baseline for all participants (Figure 1). Across all participants, excluding P7, the self-criticism records followed a pattern of consistent changes in level and trend after introduction of the treatment intervention. During the treatment phase four of five participants showed statistically significant reductions in self-criticism compared to baseline phase (Table 2), and following treatment, had scores that did not overlap with their baseline scores.

Scores on the self-judgment subscale of the SCS-SF decreased from pre- to posttreatment for all participants with 11% to 44% (Table 4). Gains were remained at follow-up for all participants except for P7.

**Table 4.** Assessment and Change Scores for Self-Compassion, Self-Criticism, Cognitive Fusion, and Psychological Flexibility.

SCS-SF									SCS-SF self-judgment subscale						
	Pre	Mid	Post	FU	Pre-post Δ (%)	Pre-FU Δ (%)	95% CI = CS ± 5.17^a^			Pre	Mid	Post	FU	Pre-post Δ (%)	Pre-FU Δ (%)
							Pre-post	Pre-FU							
P1	1.83	2.33	3	2.92	64	60	[0.68, 1.66]*	[0.6, 1.58]*	P1	4.5	4	2.5	3	−44	−33
P2	1.83	2.17	3	3	64	64	[0.68, 1.66]*	[0.68, 1.66]*	P2	5	5	3.5	4	−30	−20
P4	2.33	2.25	3	3.08	29	32	[0.18, 1.16]*	[0.26, 1.24]*	P4	5	5	4	3.5	−20	−30
P5	2.58		3.33	3.75	29	45	[0.26, 1.24]*	[0.68, 1.66]*	P5	3.5		3	2	−14	−43
P7	1.5	2	1.83	1.67	22	11	[−0.16, 0.82]	[−0.32, 0.66]	P7	4.5	4	4	4.5	−11	0
CFQ-7									BDD-AAQ						
	Pre	Mid	Post	FU	Pre-post Δ (%)	Pre-FU Δ (%)	95% CI = CS ± 7.60^a^			Pre	Mid	Post	FU	Pre-post Δ (%)	Pre-FU Δ (%)
							Pre-post	Pre-FU							
P1	47	34	19	23	−60	−51	[−35.74, 20.26]*	[−31.74, −16.26]*	P1	33	31	62	52	88	58
P2	41	35	24	30	−41	−27	[−24.74, −9.26]*	[−18.74, −3.26]*	P2	16	28	46	40	188	150
P4	35	39	35	24	0	−31	[−7.74, 7.74]	[−18.74, −3.26]*	P4	25	29	47	35	88	40
P5	44		25	28	−43	−36	[−26.74, −11.26]*	[−23.74, −8.26]*	P5	24		31	35	29	46
P7	38	41	46	44	21	16	[0.26, 15.74]	[−1.74, 13.74]	P7	25	28	32	29	28	16

*Note*. Negative change scores indicate decreases on a given measure, positive change scores indicate increases. CI = confidence interval; CS = change score; pre-post = change from pretreatment score to post-test (after Session 12); pre-FU = change from pretreatment to 6 month follow-up; P = participant; SCS = Self-Compassion Scale-Short Form; CFQ-7 = Cognitive Fusion Questionnaire—7; BDD-AAQ = Acceptance and Action Questionnaire modified for BDD.

a The 1.96 × Sdiff value used for each measure to calculate the 95% CIs around each change score.

* Indicates improvement *p* < .05.

### Shame

The daily records of body shame showed large fluctuations from day-to-day during all phases, including baseline phase (Figure 1). However, Tau-U analysis showed a statistically significant change in level of nonoverlapping scores between the treatment and baseline phases for three of the participants (Table 2). At posttreatment, four participants had scores that did not overlap with their baseline scores, with P4 and P1 demonstrating clear improvements. At follow-up phase, P4, P1, and P2 had maintained or extended their gains.

At intake, global shame measured by the ISS, was in the range of moderate to severe shame severity for all participants (*M* = 69.0, *SD* = 7.9, range 58–78). Notably, at mid treatment shame scores had further increased for three of the patients. However, following treatment, shame had reliably decreased for three of five participants and at 6 months follow-up four of the participants showed significant reductions in shame scores (25%–66%) compared to pretreatment (Table 3).

### Depression

At pretreatment, all of the participants had scores on the MADRS-S indicating moderate to severe depression (*M* = 29.0, *SD* = 4.9, range 23–35). Four of the five participants reported reliable and large decreases in depressive symptoms between pre- and posttreatment (Table 3). P7 showed a small but nonsignificant improvement. At follow-up, all participants except for P7 maintained or extended their gains, with change scores ranging from 60% to 89% compared to pretreatment, and fell in the range of mild to subclinical or no symptoms.

### Quality of Life

Before treatment, all participants reported low or extremely low quality of life, based on the QOLI (*M* = −1.24, *SD* = 2.02, range −2.88 to 1.21). After treatment, scores of quality of life had significantly and strongly improved for P1, P2, and P5 (with 167%–301%), and all participants except P7 had scores comparable to non-clinical samples in previous studies (Lindner et al., 2013). At 6-month follow-up four of five participants, were considered reliably improved. For P7 quality of life worsened during treatment phase and follow-up. During P7’s treatment phase, social restrictions were inflicted in the local community due to the outbreak of Covid-19, that made him almost completely socially isolated and reportedly contributed to lower his quality of life.

#### Process measures

See Table 4 for scores on self-report measures of self-compassion and psychological flexibility, that is, the proposed treatment processes.

### Self-Compassion

At intake, all participants had scores on the SCS-SF, measuring self-compassion, comparable or lower than previously published means for a clinical sample with eating disorders (Kelly et al., 2014). At posttreatment, P1, P2, P4, and P5 showed significant increases in self-compassion (change scores between 29% and 64%) and fell in the range of previously published nonclinical means (Kelly et al., 2014). At follow-up, the same four participants maintained or extended their gains. P7 reported an increase in self-compassion posttreatment, but the change was non-significant.

### Psychological Flexibility

All participants reported improvements in psychological flexibility measured by the BDD-AAQ, with gains ranging from 28% to 188% posttreatment. Cognitive fusion, measured by the CFQ-7, reliably decreased (with 41%–60%) for P1, P2, and P5 during treatment. For P4, scores were unchanged at posttreatment but had significantly improved at follow-up. On the contrary, P7 reported an increase in cognitive fusion posttreatment.

### Treatment Credibility and Acceptability

After psychoeducation phase, all participants rated ACTwC for BDD as credible (*M* = 8, range 7–10) and reported high expectancy of treatment efficacy for their problems (*M* = 7.25, range 6–10).

According to the CSQ-8, the four treatment responders were very satisfied with the treatment they received, whereas the non-responder reported he was indifferent or somewhat displeased. Furthermore, all treatment responders found treatment quality to be excellent, reported that it met almost all or most of their needs, that they learned a much better approach to their problems and that they would recommend ACTwC to a friend with similar problems. P7, who did not respond to the treatment, reported that treatment quality was ok but the treatment only met a few of his needs.

## Discussion

This study used a controlled multiple baseline design methodology to evaluate a 12-session acceptance and compassion-based intervention targeting shame in BDD. Our first aim, to investigate preliminary effectiveness of ACTwC for BDD, was supported. The intervention led to large improvements in BDD-behaviors and self-criticism for all but one participant, and reductions in body shame for three of five participants. The standardized outcome measures administered pre-, mid-, and posttreatment followed the same pattern as the daily measures with significant decreases for most participants in overall BDD-symptoms, self-criticism, and general shame at posttreatment. In addition, treatment led to large and reliable improvements in quality of life and depressive symptoms for all but one participant. Improvements were maintained at 6-months follow-up.

The intervention’s effect on shame and self-criticism is worth reflecting on in some more detail. Overall, self-criticism decreased faster and more consistently than shame across most subjects. Body shame fluctuated from day-to-day throughout treatment and the standardized measures even showed some increases in general shame at midpoint. However, at follow-up the level of body shame had decreased compared to baseline for three participants and general shame had significantly improved for four of the participants compared to pretreatment. The slow and uneven gains in shame is consistent with previous findings by Luoma et al. (2012), who found that a shame focused treatment resulted in slower decreases during active treatment than the control condition and improvement in shame mainly occurred at follow-up.

The temporary increases and slow gains in shame during treatment phase may be interpreted as an effect of a treatment focus on eliciting contact with previously avoided feelings of shame. The ability to acknowledge, accept, and regulate the experience of shame was promoted throughout treatment. In an ACT approach, fostering awareness and openness to undesirable thoughts and feelings (e.g., of shame), is key to reduce negative effects of inner experiences. In fact, the real goal in ACT is to promote psychological flexibility and value-guided action in the presence of painful emotions, rather than reducing emotions themselves. However, when psychological flexibility in response to difficult thoughts and feelings increases, usually the emotions are also perceived as less burdensome and tend to decrease, which may explain the long-term reductions in shame.

Even though the treatment was designed to target shame, it also had a large effect on patient’s BDD-behaviors, providing some preliminary support for the proposition by Veale and Gilbert (2014) that shame is related to BDD-behaviors. However, future research is needed to determine temporal and causal relations between interventions, shame, and BDD-behaviors.

Furthermore, the secondary outcomes showed large and reliable treatment effects on BDD-symptoms for most participants. At posttreatment, four of the five participants could be classified as treatment responders, reflecting clinically meaningful reduction on the BDD-YBOCS according to the predefined criterion, and as achieving symptom remission at follow up. The treatment response and symptom remission rates gained in this study is excellent compared to what is typically found in clinical trials of BDD. In comparison, a study by Fernández de la Cruz et al. (2021) where data from three randomized clinical trials of CBT for BDD were pooled, found that 42.67% of participants could be classified as treatment responders and only 30.67% were in symptom remission at the end of treatment.

In addition, the four responders in the present study made large and reliable improvements in quality of life and depressive symptoms. They went from moderate-severe depression at pretreatment to mild or subclinical symptoms after treatment, and from low quality of life to levels comparable to non-clinical samples in previous studies (Lindner et al., 2013). All gains were remained at 6-months follow-up for the four treatment responders. In summary, these results provide some evidence that it is feasible for people with moderate to severe BDD, elevated shame and even comorbidity like depression, to markedly improve in both symptoms and quality of life during a relatively short period of time.

Congruent with expectations, the treatment also had a significant effect on the proposed treatment processes. Most participants demonstrated increases in psychological flexibility and self-compassion after treatment, suggesting that the intervention strengthened these abilities. Additional research is needed to evaluate the impact of these proposed treatment processes. Future studies should investigate the mediating or moderating potential of these processes on the effects of shame in BDD.

The second objective of the present study was to evaluate the feasibility of this new treatment approach to BDD. Most participants reported high levels of satisfaction with the intervention and stated that it met almost all their needs. They all found the treatment to be highly credible. Hence, the second aim was supported, indicating that ACTwC was acceptable to people with BDD and elevated shame, and feasible to be delivered in a psychiatric outpatient setting.

Strengths of this study include the randomized multiple baseline design that controlled for threats to internal validity, such as the passage of time and repeated assessments. Further, the daily assessment method has the advantage that it is sensitive to change and fluctuations in mood and behaviors. Additional strengths were the real-world setting and the large improvements across patients with considerable symptoms and low quality of life. Moreover, we used three different therapists who after moderate training were able to deliver treatment adequately, providing some initial evidence that the intervention can be successfully delivered in a real-world context.

Single-case experimental designs (e.g., multiple baseline) are suitable for initial evaluation of psychological treatments (American Psychological Association presidential task force on evidence-based practice, 2006). With this design each participant acts as their own control, therefore fewer subjects are needed to demonstrate change as a result of an intervention. However, generalizability to the broader population is obviously limited due to the low number of participants. Consequently, larger randomized controlled studies are needed to confirm the results and hypotheses generated by this study.

Another limitation of the study is the relatively unknown reliability and validity of the daily assessments of body shame and self-criticism. The daily measures consisted of single item questions that required participants to be able to understand these constructs. Identifying and discriminating self-conscious emotions can be difficult (Tangney & Dearing, 2002). To address this issue, the concepts of body shame and self-criticism were carefully described and exemplified to the participants before initiating baseline phase. In addition, the standardized outcome measures of shame, self-criticism, and BDD symptoms, overall confirmed the patterns in the daily ratings, indicating that they did capture changes in the intended variables. Nonetheless, future studies should include validated measures of body shame. To date, there is a lack of instruments that accurately captures the experiences of shame in BDD. The development of new and improved measures of experienced body shame relevant in BDD, or related concepts like self-directed disgust (Stasik-O’Brien & Schmidt, 2018), would likely facilitate future research advancements in this area.

## Conclusions

In conclusion, the results from this pilot study suggest that ACT with Compassion has potential to reduce BDD symptoms, shame, and self-criticism, and in addition, improve depressive mood and patients’ quality of life. Also, the treatment was considered acceptable to the patients. Feedback and study results will be used to guide further development of the treatment protocol and future research that explores treatment moderators and mechanisms of change and evaluates efficacy of the intervention compared to other treatments in a larger randomized controlled trial.

## Supplemental Material

sj-docx-1-bmo-10.1177_01454455221129989 – Supplemental material for Acceptance and Compassion-Based Therapy Targeting Shame in Body Dysmorphic Disorder: A Multiple Baseline Study Click here for additional data file. Supplemental material, sj-docx-1-bmo-10.1177_01454455221129989 for Acceptance and Compassion-Based Therapy Targeting Shame in Body Dysmorphic Disorder: A Multiple Baseline Study by Johanna Linde, Jason B. Luoma, Christian Rück, Jonas Ramnerö and Tobias Lundgren in Behavior Modification

sj-docx-2-bmo-10.1177_01454455221129989 – Supplemental material for Acceptance and Compassion-Based Therapy Targeting Shame in Body Dysmorphic Disorder: A Multiple Baseline Study Click here for additional data file. Supplemental material, sj-docx-2-bmo-10.1177_01454455221129989 for Acceptance and Compassion-Based Therapy Targeting Shame in Body Dysmorphic Disorder: A Multiple Baseline Study by Johanna Linde, Jason B. Luoma, Christian Rück, Jonas Ramnerö and Tobias Lundgren in Behavior Modification
