# Supplementary material for: Acceptance and Compassion-Based Therapy Targeting Shame in Body Dysmorphic Disorder: A Multiple Baseline Study
Source: Behav Modif. 2022 Nov 13;47(3):693–718. doi: 10.1177/01454455221129989 (PMC10150257; doi:10.1177/01454455221129989)
Supplement: sj-docx-1-bmo-10.1177_01454455221129989 – Supplemental material for Acceptance and Compassion-Based Therapy Targeting Shame in Body Dysmorphic Disorder: A Multiple Baseline Study [file sj-docx-1-bmo-10.1177_01454455221129989.docx]

BDD-AAQ

*Directions: Below you will find a list of statements. Please rate the truth of each statement as it applies to you. Use the following rating scale to make your choices. For instance, if you believe a statement is ‘Always True,’ you would write a 7 next to that statement.*

| Never True | Very Seldom True | Seldom True | Sometimes True | Frequently True | Almost Always True | Always True |
| --- | --- | --- | --- | --- | --- | --- |
| 1 | 2 | 3 | 4 | 5 | 6 | 7 |

1. Worrying about my appearance makes it difficult for me to live a life that I value.
2. I care too much about my appearance.
3. I shut down when I feel bad about the way I look.
4. My thoughts and feelings about my appearance must change before I can take important steps in my life.
5. Worrying about the way I look takes up too much of my time.
6. If I start to feel ugly, I try to think about something else.
7. Before I can make any serious plans, I have to feel better about my appearance.
8. I will have better control over my life if I can control my negative thoughts about my appearance.
9. To control my life, I need to control my appearance.
10. Feeling ugly causes problems in my life.
11. When I start thinking about the way I look, it’s hard to do anything else.
12. My relationships would be better if my appearance did not bother me.
