# Supplementary material for: Acceptance and Compassion-Based Therapy Targeting Shame in Body Dysmorphic Disorder: A Multiple Baseline Study
Source: Behav Modif. 2022 Nov 13;47(3):693–718. doi: 10.1177/01454455221129989 (PMC10150257; doi:10.1177/01454455221129989)
Supplement: sj-docx-2-bmo-10.1177_01454455221129989 – Supplemental material for Acceptance and Compassion-Based Therapy Targeting Shame in Body Dysmorphic Disorder: A Multiple Baseline Study [file sj-docx-2-bmo-10.1177_01454455221129989.docx]

ACT with Compassion for BDD – Summary of treatment content

| Session | Theme | Overarching content | Homework |
| --- | --- | --- | --- |
| 1-2 | Conceptualization and psychoeducation | Explore patient´s appearance problems, sources of psychological rigidity and learning history (including shame and criticism).  Introduce treatment approach and form.  Psychoeducation on treatment concepts (e.g. conceptualization of BDD and shame from an ACT and compassion perspective).  Aim: Comprehensibility and de-blaming | Learning history  Practice awareness (observe own reactions) |
| 3 | Value-guided action | Identify values, barriers and formulate steps in valued direction.  Aim: Reconnect with values and promote value-guided actions in daily life | Steps in valued direction  Practice awareness |
| 4 | Emotional avoidance  -> Acceptance & self-compassion | Through experiential exercises (e.g. creative hopelessness) illustrate the paradoxical effects of emotional avoidance and control.  Aim: Create willingness and self-compassion towards inner experiences | Practice awareness  Willingness Diary  Letting go of control behaviors  Steps in valued direction |
| 5-6 | Awareness and openness | Practice to nonjudgmentally notice and observe inner experiences, including shame and self-criticism (e.g. through basic mindfulness training).  Differentiate between being aware of the present moment and being caught in own thoughts.  Conceptualize patient’s self-critical self-talk from an ACT and compassion perspective.  Build defusion and perspective taking abilities through experiential exercises.  Aim: Build skills in present-centered, flexible, nonjudgmental awareness, especially of own suffering. | Practice awareness and openness  Willingness Diary  Letting go of control behaviors  Steps in valued direction |
| 7 | Self-compassion | Identify and reconceptualize patient’s relationship to the self – show new self-to-self approach is possible.  Practice self-compassion through experiential exercises (e.g. imagery).  Aim: Foster self-compassionate self-talk. Increase behavioral repertoire of compassion towards self and others. | Practice awareness and openness  Practice self-compassion  Letting go of control behaviors  Steps in valued direction |
| 8-11 | Transforming shame | Training in acknowledging, eliciting, and regulating previously avoided emotions of shame. Practicing self-compassion in response to shame and self-criticism. Exposure to internalized shame through chair-work techniques.  Aim: Foster psychological flexibility and self-compassion in response to shame and self-criticism | Practice awareness, openness and self-compassion in response to shame and self-criticism  Letting go of control behaviors  Steps in valued direction |
| 12 | Relapse prevention | Aim: Consolidation of skills and maintenance of gains. |  |
